# Review: Magnetic Resonance Spectroscopy Studies of Pediatric Major Depressive Disorder

**DOI:** 10.1155/2011/650450

**Published:** 2010-10-04

**Authors:** Douglas G. Kondo, Tracy L. Hellem, Young-Hoon Sung, Namkug Kim, Eun-Kee Jeong, Kristen K. DelMastro, Xianfeng Shi, Perry F. Renshaw

**Affiliations:** ^1^The Brain Institute at the University of Utah, 383 Colorow Drive, Salt Lake City, UT 84108-1201, USA; ^2^Department of Psychiatry, University of Utah School of Medicine, 30 N. 1900 E, Salt Lake City, UT 84132, USA; ^3^Department of Radiology, University of Utah School of Medicine, 30 N. 1900 E, Salt Lake City, UT 84132, USA

## Abstract

*Introduction*. This paper focuses on the application of Magnetic Resonance Spectroscopy (MRS) to the study of Major Depressive Disorder (MDD) in children and adolescents. *Method*. A literature search using the National Institutes of Health's PubMed database was conducted to identify indexed peer-reviewed MRS studies in pediatric patients with MDD. *Results*. The literature search yielded 18 articles reporting original MRS data in pediatric MDD. Neurochemical alterations in Choline, Glutamate, and N-Acetyl Aspartate are associated with pediatric MDD, suggesting pathophysiologic continuity with adult MDD. *Conclusions*. The MRS literature in pediatric MDD is modest but growing. In studies that are methodologically comparable, the results have been consistent. Because it offers a noninvasive and repeatable measurement of relevant *in vivo* brain chemistry, MRS has the potential to provide insights into the pathophysiology of MDD as well as the mediators and moderators of treatment response.

## 1. Introduction

Led by a major research initiative from the U.S. National Institute ofMental Health (NIMH), the hunt is on for biomarkers in psychiatry [1]. A biomarker is a characteristic that is objectively measured and evaluated as an indicator of normal biological processes, pathogenic processes, or pharmacological responses to a therapeutic intervention [2]. The readiness of biological markers to serve as features, risk factors, or diagnostic criteria is of significant concern in the development of the DSM-V [3], and the research agenda for DSM-V emphasizes the need to translate research findings into a new classification for psychiatric disorders [4, 5]. In psychiatry, biomarkers could be used to detect and assess or to predict the development of psychiatric disorders—and more importantly, biomarkers could be used to inform treatment decisions [6]. It has been argued that the DSM-V should be structured to permit incorporation of well-replicated findings from neuroscience, by creating mechanisms to flexibly evaluate genetic markers or neuroimaging results rather than waiting for publication of the DSM-VI [7]. A consensus has emerged that advances in the assessment, treatment, and prevention of brain disorders are likely to originate from studies based on clinical and translational neuroscience [8].

In the search for these translational tools, neuroimaging is a strong candidate to aid psychiatry in its quest to join the other specialties in medicine in utilizing tests anchored in biology for delivering care and developing new interventions. Advances in child psychiatry neuroimaging have begun to provide a scientific infrastructure for understanding numerous psychiatric disorders [9, 10]. Because it can define the neural structures and pathways that mediate illness and its progression, imaging has the potential for use in clinical decision making and disease monitoring [11]. Owing to the fact that depression is not associated with gross tissue pathology or with unambiguous animal models for spontaneous and recurrent episodes, the availability of research tools to noninvasively assess the brain is critical to elucidating the neurobiology of mood disorders [12]. In addition to the scientific insight they provide, the neuroimaging methods employed in child and adolescent psychiatry are noninvasive and have proven to be safe [13]. 

Child and adolescent psychiatry investigators have adopted a number of neuroimaging approaches. The purpose of this paper is to review the findings reported using Magnetic Resonance Spectroscopy (MRS) in the study of pediatric Major Depressive Disorder (MDD). As the name suggests, MRS is a procedure that allows measurement of relevant neurochemistry. Its application to the study of MDD is therefore of particular interest, because mood disorders are illnesses of “state” [14, 15]. If MRS is able to define and validate neurochemical biomarkers, then results of MRS studies may one day be utilized to diagnose mood disorders and to monitor treatment response. 

MDD is a significant global health problem, ranking 3rd on the World Health Organization's list of the most common causes of disability worldwide [16]. In the U.S., the National Comorbidity Survey Replication study found that the peak age of onset for mental disorders is 14 years, with MDD most commonly emerging during adolescence [17, 18]. With an annual incidence of 2% in children and 4% to 8% in adolescents [19], and a cumulative lifetime prevalence of up to 20% [20], MDD in children is associated with academic failure, social impairment, substance abuse, and suicide attempts [19]. Compared with healthy controls, depressed adults are more likely to have had a depressive episode in adolescence [21]. Adding to the morbidity and mortality experienced by patients and their families, pediatric MDD imposes a substantial economic burden on society [22]. The personal and financial toll is amplified by the fact that only 50% of patients with MDD are diagnosed before reaching adulthood [23]. Therefore, novel diagnostic and treatment tools for patients in the critical adolescent stage of development are urgently needed [24–27].

The paper begins with a brief description of how MRS data is acquired, the information that is obtained, and how that information differs from the results of other neuroimaging methods. Following a review of MRS studies in pediatric MDD, the paper concludes with suggested directions for further study.

## 2. Magnetic Resonance Spectroscopy: A Primer

In 1946, Felix Bloch at Stanford University and Purcell at the Massachusetts Institute of Technology independently demonstrated the phenomenon of Nuclear Magnetic Resonance (NMR) [28, 29]. It was known that the nuclei of certain elements, for example, hydrogen (^1^H) and phosphorus (^31^P), have magnetic properties and spin. Bloch and Purcell showed that nuclei containing an odd number of nucleons (i.e., protons + neutrons) could absorb energy at a specific resonance when placed in a strong magnetic field. Based upon the principles of NMR, magnetic resonance imaging (MRI) was introduced to clinical medicine in the 1970s [30]. The principles of NMR were used to develop magnetic resonance spectroscopy (MRS), which enabled characterization of living tissues based upon their chemical constituents [31]. Whereas MRI depicts the spatial distribution of protons of water, which are transformed into a visual representation of anatomy for interpretation, MRS detects various molecules present at concentrations on the order of mM (millimolar) [32]. For a review of the scientific principles underlying MRS, the interested reader is referred to the referenced publications [33–36].

**Figure 1:**
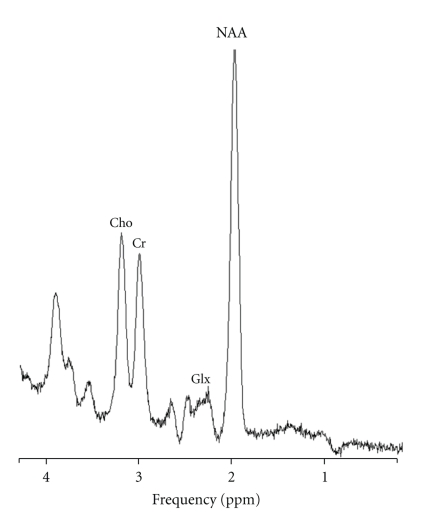
Representative proton (^1^H) magnetic resonance spectrum of the anterior cingulate cortex at 3 Tesla (TR = 2000 ms, TE = 135). Cho = Choline; Cr = Creatine; Glx = Glutamine + Glutamate; NAA = N-Acetyl Aspartate; ppm = parts per million.

The key distinction between MRI and MRS is the type of information the magnetic resonance signal is used to encode. MRI studies create anatomical images whereas MRS provides *quantitative* biochemical information about the tissue under study. Rather than high-resolution images, MRS data are presented as graphical spectra, with the area under each peak representing the relative concentration of nuclei detected for a given atomic species, for example, hydrogen or phosphorus. The *x*-axis of graphed MRS data denotes the frequency shift localizing the metabolite in parts per million. MRS spectra peaks correspond with specific chemical compounds of interest. Thus, MRS non-invasively provides a repeatable measure of chemical concentration data in living tissues, including the human brain. For the patient, the procedure is identical to a clinical study except for the amount of time spent in the scanner; the duration ranges from less than 10 minutes for acquisition of proton spectra to approximately 30 minutes for a phosphorus scan. MRS scans use no radiation, which allows for repeated measurement at different times in the course of a patient's illness, for example, prior to the start of treatment and at the point that remission is achieved. Figure 1 shows an example of ^1^H-MRS spectrum obtained from the anteriorcingulate cortex of a patient with MDD.

The chemicals quantifiable with ^1^H-MRS include the following: N-Acetyl-Aspartate (NAA), creatine (Cr), choline (Cho), myoinositol (mI), and lactate (Lac). The term “GLX” (Glx) is used to designate the single peak containing the amino acid neurotransmitters Glutamate (Glu), Gamma-Aminobutyric Acid (GABA), and Glutamine (Gln) [37], because ^1^H-MRS signals from Glu and Gln are complicated by the interaction of neighboring protons and the pH dependence of chemical shift [38]. Glu is the major excitatory neurotransmitter in the human brain and was first measured in 1992 [39]. Brain *in vivo* concentrations of Glu are approximately 8–13 times that of GABA, and the ratio of Glu/Gln ranges from 2.4–3.8; therefore, alterations in Glx are typically attributed to altered Glu concentrations [40]. GABA, the major inhibitory neurotransmitter in the brain, has an ^1^H-MRS peak that can be separated from Glx at magnetic field strengths ≥2 Tesla using spectral editing technique or 2-D J-resolved spectra [41]. 

NAA is the most prominent ^1^H-MRS peak and is found only in the nervous system [42]. It is a marker of neuronal density or function, osmoregulation, and energy homeostasis; there is a direct relationship between NAA synthesis, oxygen consumption, and ATP production in the central nervous system [43]. NAA may also play a critical role in myelin production within oligodendrocytes [44]. Reduction in NAA levels measured by ^1^H-MRS is a recognized marker of neuronal loss or dysfunction in several psychiatric and neurological disorders including drug abuse, schizophrenia, traumatic brain injury, stroke, epilepsy, multiple sclerosis, neoplasm, HIV encephalopathy, and Alzheimer's disease [43]. 

The Cr peak reflects the sum of the Cr and Phosphocreatine (PCr) peaks, an important limitation that will be discussed below. The equilibrium maintained between Cr and PCr is determined by the cellular demand for the high-energy phosphate stored as creatine phosphate [45]. As its level is considered to be constant, Cr is often used as an internal standard for comparison [46]. The Cho peak contains four membrane- and myelin-related chemicals [36]: phosphorylethanolamine (PE), phosphorylcholine (PC), glycerophosphorylethanolamine (GPE), and glycerophosphorylcholine (GPC). Cho is a metabolic marker of membrane density and integrity, that is, phospholipid synthesis and degradation [30]. Neuropathology characterized by cell membrane breakdown liberates Cho and increases the free Cho pool, contributing to an increased resonance in neurodegenerative disorders [45]. In traumatic brain injury, Cho levels increase in relation to the severity of neuronal injury resulting from the breakdown of membranes and myelin [47]. Finally, an elevation in the Cho resonance within brain lesions has been accepted as a sign of malignancy [48, 49]. Myoinositol (mI) is a sugar involved in the regulation of neuronal osmolarity, the metabolism of membrane bound phospholipids, and in the phosphoinositide (PI) secondary messenger pathway [46]. Myo-inositol is considered a marker of glial proliferation, and an increase in mI resonance may be a proxy for increased inflammation in the brain [50]. Under normal circumstances, lactate (Lac) is present in the brain at concentrations too small to be detected using ^1^H-MRS. However, if the aerobic oxidation mechanism fails and anaerobic glycolysis is triggered—such as brain ischemia, hypoxia, seizure activity, and metabolic disorders—Lac levels rise significantly [30]. Typically, the Lac peak can be observed as an inverted doublet at an echo time of 135 ms at 1.3 ppm. It has been shown that lactate becomes elevated if large numbers of inflammatory cells are activated [51]. In the past two decades, ^1^H-MRS has progressed from the laboratory into routine use in the treatment of cancers of the brain and prostate [33]. Mood disorders may join the list of disease states in which clinicians can make use of ^1^H-MRS, if the current pace of neuroimaging research in psychiatry is maintained.

^31^P-MRS is a related neuroimaging method that acquires the resonance spectra of phosphorus rather than hydrogen. Although MRS can be performed on a variety of nuclei such as carbon, nitrogen, fluorine, and sodium, only the nuclei of phosphorus (31P) and hydrogen (1H) exist *in vivo* in sufficient concentrations for routine clinical evaluation [52]. Studies employing ^31^P-MRS have indicated possible abnormalities in membrane high-energy phosphate metabolism, phospholipid metabolism, and intracellular pH in mood disorders [32]. 

**Figure 2:**
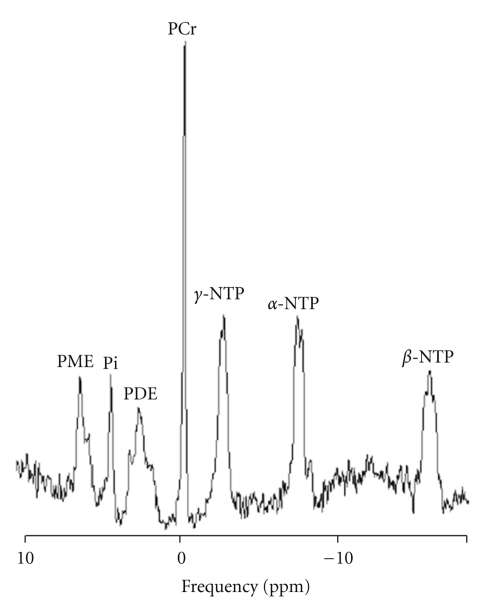
Phosphorus (^31^P) magnetic resonance spectrum of the whole brain at 3 Tesla (TR = 3000 ms, TE = 2.3 ms). PME = phosphomonoester; Pi = inorganic phosphate; PDE = phosphodiester; PCr = phosphocreatine; NTP = Nucleoside Triphosphate; ppm = parts per million.

In ^31^P-MRS spectra of the brain, seven chemical peaks are resolved; these are phosphomonoester (PME), inorganic phosphate (Pi), phosphodiester (PDE), phosphocreatine (PCr), and alpha-(*α*), beta-(*β*), and gamma-(*γ*) nucleoside triphosphate (NTP) [32, 53, 54]. Figure 2 displays an example of thespectra that is acquired.

The *β*-NTP peak is measured as a proxy for ATP, the principal energy source in brain. The phosphomonoester (PME) peak contains the signals from numerous metabolites, including those related to membrane phospholipid synthesis such as phosphocholine (PC) or phosphoethanolamine (PE) [55] and sugar phosphates such as glycerophosphate or inositol phosphates [56]. In the PME region, PE is the most abundant and PC is the second most abundant metabolite [57]. The membrane breakdown products glycerophosphocholine and glycerophosphoethanolamine contribute to the PDE peak [58], but most of the signal in the *in vivo* PDE peak arises from membrane phospholipid itself [59, 60], marking PDE as a marker of neuronal integrity. The Pi peak appears between the PME and PDE peaks. Pi appears in many metabolic pathways. Although the Pi peak contains both PO_4_^−1^ and PO_4_^−2^, they register as one peak due to the rapid exchange between these two molecules. The position of this peak reflects the equilibrium between PO_4_^−1^ and PO_4_^−2^, a fact that allows investigators to calculate brain pH from the chemical shift of the Pi peak [61]. Because the phosphate ions exist in the intracellular space, this calculated pH reflects intracellular pH (pHi). The PCr peak is the most prominent peak in the ^31^P-MRS spectra in the brain [32]. PCr conveys high-energy phosphates from the mitochondria to the cytosol. When an ATP molecule is consumed, PCr transfers its high-energy phosphate group to ADP (adenosine diphosphate), thus replenishing ATP via the creatine kinase reaction. In this regard, PCr behaves as a buffer of ATP [62]. PCr is abundant in tissues with rapidly variable energy demands, that is, brain and muscle tissue. This ATP buffer is absent in tissues where energy demands are constant, such as liver tissue. NTP forms three distinct peaks—alpha-(*α*), beta-(*β*), and gamma-(*γ*) nucleoside triphosphate—of which the doublet of the *γ*-ATP peak is resolved in 31P-MR spectra [32]. ATP is the bioenergetic substrate for many biochemical processes in the brain and is present at a much higher concentration—on the order of 1.8 mM—than any other NTP [63]. 

For a number of practical reasons, proton spectroscopy is the most widely employed MRS method in psychiatric research. Protons are abundant in organic structures, and their nuclei have high magnetic sensitivity. In 1995, ^1^H-MRS became widely available when the U.S. Food and Drug Administration (FDA) approved the software for an automated and inexpensive MRS sequence protocol, the PROton Brain Examination (PROBE) [31, 64], which can be run without dedicated research personnel on a standard MRI scanner [31]. The majority of ^1^H-MRS studies have been conducted using MRI scanners operating at magnetic field strengths of 1.5 Tesla or less, which is less than optimal for ^31^P-MRS. However, 3 Tesla MRI was approved by the FDA in 2000 [65] and is becoming widely accessible to major medical centers. The increased availability of scanners with 3 or 4 Tesla magnetic fields will improve the sensitivity of MRS studies [66]. At present, to conduct MRS brain scans that target other nuclei of interest to psychiatrists—such as phosphorus or lithium—requires specialized equipment (though not a separate MRI machine!) and research expertise. These obstacles will not be insurmountable, if ^31^P-MRS, in particular, proves to be a valid and reliable measure of one or more translational biomarkers in the affective disorders. To date, unlike physicians in other fields of medicine, psychiatrists do not benefit from working with objective measures of illness and recovery, such as blood pressure or hemoglobin A1c. In the competition to define the first validated biomarker for use in clinical psychiatry, MRS investigators have joined the pursuit along with their colleagues in genetics, neuroscience, and other branches of neuroimaging.

In recent years, multiple studies have reported regional and global hypometabolism in subjects experiencing a major depressive episode, which could be related to the pathophysiology of mood disorders [67]. The literature describes several abnormalities of bioenergetic metabolism in adults, primarily decreased baseline levels of *β*-nucleoside triphosphate and total NTP, in the basal ganglia and the frontal lobes of MDD subjects compared with healthy control subjects [63, 68, 69]. Our group recently reported baseline PCr levels could be a predictor of depression treatment outcomes [67]. Thus, ^31^P-MRS provides investigators with a robust methodology [70], with which it is now possible to test specific hypotheses regarding the neurobiology of mood disorders in adults. MDD is a common and disabling illness that often begin in adolescence [23, 71–73]. Given the well-established safety of MRI, further study of pediatric mood disorders utilizing ^31^P-MRS represents a rational endeavor in an attempt to expand the evidence base in medicine.

## 3. Materials and Methods

### 3.1. Search Strategy

A literature search using the U.S. National Library of Medicine's PubMed database was conducted to identify peer-reviewed neuroimaging MRS research studies of children and adolescents with MDD that were published between January 1966 to March 2010. The following terms were included in the search: “magnetic resonance spectroscopy,” “depressive disorder,” “mood disorder or affective disorder,” and “child or adolescent or pediatric or early-onset.” We performed a backward search of bibliographic references from the identified articles to ensure the inclusion of relevant articles. A forward citation search for identified studies was also performed. Studies that recruited a mixture of adults and children were not included. All relevant articles published in English were included, and due to the small number of studies no methodological exclusion criteria were applied.

## 4. Results and Discussion

The literature search yielded 18 articles reporting original MRS data in pediatric MDD: 13 studies in MDD and 5 in children and adolescents with mood disorders not meeting full diagnostic criteria for MDD.  Table 1 presents the published MRS studies in pediatric MDD, all of which employed ^1^H-MRS. 

Following up on their report of structural abnormalities in the brains of children hospitalized for depression, Steingard and colleagues reported in 2000 finding increased choline/creatine ratios and increased choline/NAA ratios in the left orbitofrontal cortex of adolescents with MDD compared with controls [74]. This suggested that brain cytosolic choline may be increased in depressed adolescents, independent of corresponding structural changes, results that were consistent with studies in adults with MDD. This report was followed in 2001 by Kusumaker's finding of decreased choline/creatine ratios in the left amygdala [75], which again implicated choline in the pathophysiology of pediatric MDD. In 2002, Farchione et al. studied the left and right dorsolateral prefrontal cortex in medication-naïve adolescents with MDD and healthy controls. A significant increase in choline was observed in left—but not right—dorsolateral prefrontal cortex in MDD patients versus controls (32.5% higher) [76]. In a three-armed ^1^H-MRS study, Smith et al. compared medication-naïve MDD patients with Obsessive-Compulsive Disorder (OCD) patients and healthy controls. Following up on a prior finding of choline alterations in OCD, the investigators studied the thalamus and found increased choline concentrations bilaterally in the medial thalamus in pediatric OCD patients compared with both MDD patients and controls; they found no difference in medial thalamic choline between MDD patients and control subjects [77]. The potential of MRS in pediatric mood disorders was confirmed by these initial studies in pediatric MDD, which joined studies in adult MDD and preclinical animal research in implicating choline alterations in the disorder [78, 79].

Two pediatric MDD ^1^H-MRS studies were published in 2004. Mirza et al. compared medication-naïve patients with healthy controls and found a 19% decrease in glutamatergic (Glx) concentrations in the anterior cingulate cortex in the MDD patients [80]. A second study of the anterior cingulate cortex found lower Glx concentrations in both MDD patients (18.7% decrease) and OCD patients (15.1% decrease) compared with healthy controls [81]. In the following year, two additional studies were published. Rosenberg et al. separated the Glutamate and Glutamine peaks and found 23% lower Glutamate concentrations in the anterior cingulate cortex of MDD patients compared with controls (*P* = .0002) [82]. Caetano and colleagues conducted a case-control study of the left dorsolateral prefrontal cortex and reported decreased choline-containing compounds and increased myoinositol concentrations in patients with MDD [83]. MacMaster andKusumakarreported results of their ^1^H-MRS study of the right prefrontal cortex in 2006,finding that choline/creatine ratio was elevated in MDD patients compared with healthy controls [84]. That same year, Mirza et al. published a study comparing patients with OCD with MDD and control subjects. The OCD patients demonstrated increased choline/Phosphocreatine concentrations in the left and right medial thalamus [85].

In recent years, investigators have expanded the range of structures and metabolites under study. Gabbay et al. reported in 2007 that adolescents with MDD had increased concentrations of both choline and creatine in the left (but not the right) caudate nucleus [86]. In 2008, MacMaster et al. were the first to report alterations in NAA in pediatric MDD, finding a 27% decrease in NAA in the left medial temporal cortex in affected subjects versus healthy controls [87]. Finally, Gabbay and colleagues obtained proton spectra with a 3 Tesla scanner and produced findings that begin to parse the subtypes of pediatric mood disorders on a neurobiological level by focusing on immune system dysregulation in pediatric MDD [88]. The investigators reported that in patients with melancholic features, plasma Kynurenine levels were positively correlated with choline concentrations in the right caudate nucleus and plasma 3-hydroxyanthranilic acid, a neurotoxic intermediate of the Kynurenine Pathway, was positively correlated with left putamen total choline [89].

Taken together, the consistency of findings in pediatric MDD validates the utility of MRS as a translational tool for studying pediatric MDD and adds to the converging lines of evidence suggesting that pediatric MDD is continuous with adult MDD [90].

## 5. Conclusions

A decade ago, Hendren et al. reviewed the neuroimaging literature in child and adolescent psychiatry and called for researchers to “identify clear structure/function hypotheses when studying childhood mental disorders that use but go beyond DSM diagnoses [91].” Echoing this sentiment, the research agenda for DSM-V emphasizes the need to translate findings from clinical neuroscience research into a new classification system based upon pathophysiology and etiological processes [92, 93]. As it continues to mature as a method, MRS is positioned to make a contribution to this evolution in psychiatry. *In vivo* MRS is the only noninvasive imaging technique capable of directly assessing the living biochemistry in localized brain regions [94]. Studies of depressed children and adolescents have a number of advantages compared with studies of adults: the effects of statistical covariates such as repeated episodes, duration of illness, multiple medications, and normal aging are avoided. Thus, MRS may be a translational research tool capable of partially obviating the developmental and environmental confounders that have made research in child psychiatry a difficult challenge.

Researchers working with pediatric MDD populations have reported MRS findings that implicate Glx, NAA, and choline (including its correlation with immune system metabolites) in the neurobiology of MDD. Altered levels of choline would be consistent with altered neural plasticity as well as animal models of depression and antidepressant response [95]. No study identified in our literature search used repeated measures MRS scans to ascertain if there are changes in neurometabolite concentrations when a child with MDD responds to treatment, that is, whether the baseline differences in Glx, NAA, and choline are differences of “state” or “trait”. Pavuluri and Sweeney have argued for the importance of obtaining functional neuroimaging measures before and after pharmacologic intervention [9], and the feasibility of this approach is demonstrated in several published studies of depressed adults [67, 96, 97]. Results from such studies will shed light on the mechanisms by which antidepressants work and may provide new treatment targets for drug development [13, 98]. In addition to pointing toward the *mechanisms* of illness recovery (i.e., the “mediators” [99]), Pavuluri also emphasizes the potential for neuroimaging studies to identify *predictors* of treatment outcome [9] (i.e., the “moderators” [99]). Identification of the mediators and moderators of pediatric MDD treatment would serve the NIMH's stated goal of moving toward personalized care.

Research in mood-disordered adults [67, 68, 100–103], combined with preclinical animal studies of depression [104–107], has given rise to the bioenergetic hypothesis, which proposes that altered energy metabolism is a reversible correlate of pathogenesis of mood disorders [108–110]. Converging lines of evidence from electron microscopy, gene expression, genotyping, and sequencing studies implicate mitochondrial dysfunction in MDD [111]. Neuroimaging studies using MRS have convincingly demonstrated that MDD is associated with mitochondrial dysfunction [73, 77–79, 112]. More specifically, a growing body of research shows that MDD subjects have decreased beta nucleoside triphosphate (b-NTP) and increased levels of phosphocreatine (PCr) compared with healthy controls [73, 78, 79]. Furthermore, successful treatment of MDD with antidepressants is associated with normalization in both NTP and PCr levels [79, 112].

Contemporary understanding of the neurobiology of depression is focused on imbalances in neural circuits [12], cellular plasticity and resilience [113, 114], and impaired neurotrophic signaling cascades [115] (for an excellent review, see Carlson et al.[116]; for the conceptual framework, see and Manji et al. [117] and Duman et al. [118]). As a research tool, MRS is unique in its ability to perform *in vivo* quantification of the neurometabolite indicators of neuronal integrity, mitochondrial functioning, cellular membrane turnover, and signaling cascades [66]. Finally, MRS is the only known method for *in vivo* measurement of gamma-aminobutyric acid (GABA) concentration [41, 119], which plays an increasingly central role in our conceptualization of mood disorders [120–123]. As the field matures, it has the potential to play a major role in delineating the neurobiology of MDD.

### 5.1. What Are the Limitations of MRS?

While MRS studies have the potential to provide unique insights into the neurobiology of pediatric MDD, technical limitations must be acknowledged. As with all neuroimaging modalities, the number of patients enrolled in a given study tends to be small. Comparison between studies (and therefore replication of key findings) is difficult, due to differences in spectra acquisition protocols and the fact that calculation of the area under a given spectral peak is open to considerable interpretation [36]; the optimal method for quantification of neurochemicals has yet to be determined. In addition, the generalizability of MRS studies is limited by potential confounds such as medication effects, duration of illness, comorbidity, and gender [124]. At present, MRS has less temporal and spatial resolution compared with MRI and functional MRI (fMRI) [125]. The volume of the brain—called the “voxel”—that is sampled in a typical ^1^H-MRS study is ≤8 mL (larger for ^31^P-MRS scans). This is problematic in the light of current models of depressive disorders, which suggest the presence of altered interactions between hierarchically distributed neural networks which are widely distributed throughout the brain [126]. Another issue is the relatively small number of metabolites that can be assayed using MRS. “Key players” such as dopamine, serotonin, and norepinephrine are not visible to an MRS scanner [38]. Finally, scanning times are relatively long (up to 90 minutes), which increases the burden on research participants and limits patient acceptance.

### 5.2. What Would a Child Psychiatrist Want to Know about MRS?

With the current emphasis on translational research in medicine, the question of science's relevance to practicing clinicians is ever-present. Busy child and adolescent psychiatrists are faced on a daily basis with questions that further study utilizing MRS has the potential to answer.

- *How can we distinguish bipolar disorder (BD) from MDD*? Chang et al. posed the question of whether neuroimaging will be used to diagnose BD in a 2006 publication, concluding that use of MRS variables such as mI or NAA for diagnosis is problematic at present, due to the variable of mood state and the lack of standardized methods for performing and interpreting the scans [112]. If children with both BD and MDD were followed and scanned longitudinally, MRS would be able to test the hypothesis that the major depressive episodes experienced by both groups of patients have the same neurochemical basis.

(ii) *What is a problem of state versus a problem of trait*? The ability of MRS to measure neurochemical changes that parallel changes in patients' clinical presentation is now well established in children and adults. In addition, further study of children and adolescents would help determine whether differences in depressed patients are the result of altered development across the life span, or if they can be documented early in development, serving as a risk factor for which prevention strategies might be employed [74].

(iii) *Can MRS help clinicians assess the effects of medication*? Our literature search did not find any publications utilizing MRS to measure changes in the brain in pediatric MDD treatment studies.

(iv) *What is the specificity of MRS in mood disorders? *The results thus far suggest that MRS can find neurochemical differences between mood disorders and OCD [77], Intermittent Explosive Disorder [127], and Attention-deficit Hyperactivity Disorder (ADHD) [128].

An illustrative example of the potential for *specificity* offered by MRS is the case of Glx and ADHD. Investigators have found elevated Glx in brain regions of interest in ADHD patients compared to healthy controls.Duman et al. [118] found increased Glx in the right prefrontal cortex and striatum of ADHD subjects, Courvoisie [119] documented increased Glx in the left and right frontal lobes, and Klempan et al. [120] found elevated Glx concentrations in the striatum treatment-naïve ADHD patients. In addition, Carey et al. have shown that changes in glutaminergic tone occur with ADHD treatment [129, 130]. In contrast to the findings in ADHD, Rosenberg [81] and Mirza [80] have shown that Glx is reduced in MDD, and Moore et al. [128] found that pediatric Bipolar Disorder with and without ADHD is differentiated by Glx concentrations in the anterior cingulate cortex.

### 5.3. What Should Future MRS Studies in Pediatric Mood Disorders Look Like?

Some twenty years ago, Bottomley enumerated the “trouble with spectroscopy papers” [131], lamenting the fact that a research tool with such precision had given rise to a medical literature whose findings are not easily reconciled. Noting the lack of standards for the conduct and reporting of clinical MRS research, Bottomley recommended that authors provide objective, rigorously quantified results and that spectra acquisition protocols be described in sufficient details that experiments could be reproduced [131]. More recently,Kreis [132] and Taylor [133] have argued cogently for the field of clinical MR spectroscopy to establish a set of scientific and quality assurance guidelines. This process is underway in oncological neuroradiology [134–136], but widely accepted standards have yet to be established in psychiatry. As shown in Table 1, the MRS literature in pediatric MDD presents the reader with a diversity of techniques, anatomical reference points, and neurochemical findings. Future studies would benefit from standardized protocol design and data reporting: as Leibenluft [13] has observed, psychiatric neuroimaging articles can be “confusing, tedious to read and…boring”—even for researchers in the field. For MRS studies of mood disorders to become a truly *translational *body of research, investigators will need to communicate their findings to the wider psychiatric community in a clear and intuitive manner; establishment of an expert consensus regarding best practices in MRS research and reporting would be a significant early step toward that goal.

Two important gaps in the medical literature were identified by the PubMed search on which this paper is based. The first is that there were no peer-reviewed publications reporting ^31^P-MRS data in pediatric MDD. ^31^P-MRS has the unique ability to measure high-energy phosphorus metabolites *in vivo* [63, 68, 69, 137], which is important because dysfunction in neuronal energy metabolism may be one mechanism of depression [109]. Second, no pediatric MDD clinical trials were identified in which MRS brain scans were used as a repeated measure, that is, performed pre- and posttreatment in order to learn which, if any, MRS metabolites were altered by the study treatment intervention. In contrast to their absence in the pediatric MDD literature, repeated measures MRS scans have often been incorporated into treatment studies of pediatric Bipolar Disorder [138–141] and related conditions [142, 143]. Incorporating ^31^P-MRS methodology and repeated measures study design into pediatric MDD clinical trials could provide fundamental insights into the neurobiology of MDD.

In conclusion, MRS is an emerging translational research tool for the study of pediatric MDD. The bulk of published MRS depression studies have been done in adult populations, but mood disorders often begin in adolescence and there is much to be learned by studying young people who are early in the course of the disorder. The non-invasive nature and relative safety of MRS make this possible. Well-designed future MRS studies in pediatric MDD will shed light on the neurobiology of depression, help to define the physiologic mechanisms illness and recovery, and identify treatment targets for the development of new interventions.

## Figures and Tables

**Table 1:** Magnetic resonance spectroscopy studies of pediatric major depressive disorder.

Study	Sample	Field strength	Voxel size & location	Findings
Caetano, et al. 2005 [83]	-14 MDD (mean age 13.6)-22 Healthy Controls (mean age 13.3)	1.5 Tesla	8cc in the Left Dorsolateral Prefrontal Cortex	*⇓* glycerophosphocholine + phosphocholine (GPC + PC; or choline-containing compounds) in MDD patients. *⇑* Inositol and myo-Inositol levels in MDD patientsIn MDD patients Glutamate level is inversely correlated with Duration of Illness as well as Number of Episodes
Farchione et al. 2002 [76]	-11 treatment-naive MDD patients (mean age 14.3)-11 healthy controls (mean age 14.3)	1.5 Tesla	0.8 cc in the Left and Right Dorsolateral Prefrontal Cortex	*⇑* Cho was observed in left (but not right) DLPFC in MDD patients versus healthy controls (32.5% higher) No difference in NAA or Cr were observed between case-control pairs
Gabbay et al. 2009 [89]	-7 MDD patients (mean age 16.2) with Melancholic Features-7 patients (mean age 16.9) with non-melancholic, reactive MDD-6 healthy comparison patients (mean age 16.2)	3 Tesla	10 cm anterior–posterior (AP) × 7 cm left–right (LR) × 6 cm inferior–superior (IS) = 420 cm3 in the Left Putamen and Right Caudate	No significant correlations were found in the healthy control group or in the MDD group as a wholeIn Melancholic patients, plasma Kynurenine concentrations were positively correlated with Right Caudate total CholineIn Melancholic patients, plasma 3-hydroxyanthranilic acid (3-HAA), a neurotoxic intermediate of the Kynurenine Pathway) was positively correlated with Left Putamen total Choline
Gabbay et al. 2007 [86]	-14 adolescents(mean age 16.2 years) who had symptoms of MDD for 8 weeks and a score ≥40 (mean=63.6) on the Children's Depression Rating Scale—Revised-10 healthy comparison patients	3 Tesla	16 (anterior-posterior) × 16 (left-right) voxels, each anominal 0.75 cm^3^in the left and right caudate, putamen and thalamus	Adolescents with MDD had significantly ***⇑*** concentrations of choline (2.11 mM versus 1.56 mM) and creatine (6.65 mM versus 5.26 mM) in the left caudate.No other neurochemical differences were observed between groups.
Kusumakar, et al. 2001 [75]	-11 MDD (mean age 16.7)-11 Healthy Controls (mean age 16.6)	1.5 Tesla	Multivoxel: 6x6 placed in anterior medial temporal region (amygdala)	*⇓* Left amygdala Cho-Cr ratios in MDD patients compared with controlsLeft amygdala NAA-Cr and right amygdala Cho-Cr and NAA-Cr did not differ significantly between patients with depression and control patients.
MacMaster et al. 2008 [87]	11 MDD, 11 Healthy Controls	1.5Tesla	0.8 mL voxel in the left and right Medial Temporal Cortex	*⇓* N-acetyl-aspartate in theleft medial temporal cortex (27%) in MDD patients versus healthy controls
MacMaster and Kusumakar, 2006 [84]	-12 MDD-11 Healthy Controls (10-18 y/o; 7 females and 5 males per group)	1.5 Tesla	4cc in the right prefrontal cortex	*⇑* Right prefrontal cortex Choline/Creatine ratio in MDD compared with healthy controls (*p* = .007)
Mirza et al. 2004 [80]	-13 psychotropic-naïve with MDD (mean age 15.5)-13 healthy controls (mean age 15.4)	1.5 Tesla	2 × 1.5 × 1 cm = 3 cc volume centered on the anterior cingulate cortex	Anterior cingulate glutamatergic (Glx) concentrations were significantly *⇓* (19% decrease) in MDD patients versus controls (9.27 +/- 0.43 versus 11.47 +/- 0.26, respectively, *p* = .000)
Mirza et al. 2006 [85]	18 pediatricpatients with major depressive disorder 9 to 17 years of age, 18 case-matched healthy controls, and 27 patients withobsessive-compulsive disorder 7 to 16 years old	1.5 Tesla	0.8 mL voxel in the medial thalamus	*⇑* left and right medial thalamiccreatine-phosphocreatine concentrations in patients with OCD compared with both healthy controls and patients with MDDCreatine-phosphocreatine concentrations did not differ significantly betweenpatients with MDD and healthy controls
Rosenberg et al. 2005 [82]	14 MDD patients (man age 15.6)14 healthy controls (mean age 15.5)	1.5 Tesla	2 × 1.5 × 1 cm = 3cc centered on the Anterior Cingulate Cortex	*⇓* anterior cingulate glutamate in MDD patients compared with controls (*p* = .0002; 23% decrease) No difference in anterior cingulate glutamine
Rosenberg, et al. 2004 [81]	-14 psychotropic-naïve patients with MDDwithout OCD (mean age 15.6)-20 non-depressed, patients with OCD (mean age 11.4)-14 healthy controls (mean age 15.5)	1.5 Tesla	2 × 1.5 × 1 cm = 3mL in the Anterior Cingulate Cortex	*⇓* Anterior cingulate glutamatergic (Glx) concentrations were significantly in OCD patients (15.1% decrease) and MDD patients (18.7% decrease) compared with controls (*p* = .002)
Smith et al. 2003 [77]	-18 drug naive outpatients with MDD without OCD (mean age 14.4)-27 drug-naive patients with OCD (mean age 10.3)-18 healthy controls (mean age 14.4)	1.5 Tesla	0.8 mL voxel in the left and right medial and lateral thalamus	*⇑* left and right medial thalamic Choline concentrations in OCD patients compared with both healthy controls and patients with MDD
Steingard, et al. 2000 [74]	-17 MDD patients (mean age 15.8)-28 healthy controls (mean age 14.5)	1.5 Tesla	15 mm x 15 mm x 15 mm (3.38cm^3^) voxel in the Left Orbitofrontal Cortex	*⇑* Choline/Creatine ratio in MDD compared with controls***⇑*** Choline/NAA ratio in MDD compared with controls
